# Redesign your in‐person course for online: Creating connections and promoting engagement for better learning

**DOI:** 10.1002/ece3.6844

**Published:** 2020-09-29

**Authors:** Nicole A. Theodosiou, Jeffrey D. Corbin

**Affiliations:** ^1^ Department of Biological Sciences Union College Schenectady NY USA

**Keywords:** core concepts, group work, online teaching, reflective writing, student engagement

## Abstract

This spring, instructors moved their courses online in an emergency fashion as campuses were closed due to the pandemic. As colleges prepare for the next academic year, there is a need to provide flexible instruction that is more intentional for quality online learning. We taught two undergraduate courses online for the first time this spring and surveyed our students’ reactions to the course experiences. From our experiences and student feedback, we identified design elements and activities that were beneficial in promoting student engagement, sense of connectivity, and learning. We describe four qualities for a successful transition to online learning: (a) big questions and core concepts; (b) peer groups including reflective writing; (c) outreach to broader scientific community; and (d) instructor's social presence in the class. Our experience gives us confidence that courses can be redesigned for online without compromising rigor or essential learning goals.

## Graphical abstract

In this manuscript, we describe four qualities for a successful transition to online learning: (a) big questions and core concepts; (b) peer groups including reflective writing; (c) outreach to broader scientific community; and (d) instructor's social presence in the class. Our experience is based on teaching two undergraduate courses fully online this spring and student feedback we received through surveys conducted at the conclusion of the term. We are confident that with thoughtful redesign, in‐person courses can be successfully converted to the online platform without compromising rigor or essential learning goals.

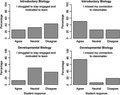

## 1 INTRODUCTION

As colleges and universities closed in mid‐March 2020, faculty had to quickly move content that was planned for in‐person teaching to an online platform. The remaining class experience followed a model referred to as (emergency) remote learning (Craig, 2020). Understandably, this emergency shift prevented instructors from undertaking the careful design process required for proven high‐quality online learning (Branch & Dousay, 2015). As colleges’ and universities’ efforts to prevent COVID infections limit classroom densities through 2020 and beyond, online learning and hybrid course models are becoming a new normal. Yet, students, families, and faculty should reasonably expect that remote instruction and learning will improve (Maloney & Kim, 2020). While successful online courses require unique tools and strategies that differ in key ways from in‐person teaching, many of the topics and recommendations discussed apply broadly to any mode of course delivery (Branch & Dousay, 2015).

Among the major challenges to designing a course, especially one that is online, are creating experiences where students feel connected to each other, and creating assignments and activities that engage students and keep them motivated to learn. In this paper, we focus on three major elements that we found promote connection and engagement among students—elements that fundamentally shape the online classroom environment: (a) collaborative learning through peer group work; (b) outreach to the broader scientific community; and (c) high‐value, personalized interactions with students. Motivation for learning is built when students are engaged with their peers and the broader scientific community. The presence of a faculty member that communicates effectively, listens to students, remains flexible, and interacts with them on different levels fosters engagement in course material. Whether designing a course for online or in‐person, we argue that well‐designed courses should refocus content and assessment from detailed‐heavy content requiring memorization to broader key concepts requiring understanding, and include original‐thinking‐based evaluations (Hodges et al., 2020).

Our experience in spring 2020 was in two undergraduate classes, both of which were exclusively remote due to the COVID‐mandated shutdown of our institution prior to the beginning of our 10‐week term. One course was an *Introductory Biology* class with an enrollment of 43 mostly first‐ and second‐year students, covering heredity, evolution, and ecology. The second class was *Developmental Biology*, a biology and biochemistry elective for juniors and seniors with an enrollment of 24 students. While both classes included a weekly laboratory component, we do not, here, detail suggestions for designing laboratory experiences for an online environment.

All class resources including content delivery, communication, assignments, and assessment were delivered via Zoom, course learning management software (LMS), a collaborative textbook reading app (Perusall), and email, among other platforms. Our recommendations are based on the feedback we have received from students and are backed by pedagogical best practices. We believe they can apply to courses that are exclusively online or courses that offer blended delivery models. Many of the tools discussed are ones that we have utilized in our own in‐person classes. While concept‐based tools for collaborative learning require thoughtful planning during course design, assessment of the activities and peer group work can be significantly less onerous than a course whose focus is on content assessment. Thus, we believe our recommendations can be adapted and scaled to courses with larger class sizes.

In order to assess student perceptions of the transition to online learning and the effectiveness of the tools we describe, we conducted a voluntary survey of each class during the final days of the spring 2020 term (Panel 1). Student responses give us assurance that the following tools can be applied to either online or hybrid learning so that few, if any, learning goals have to be compromised as instructors and institutions navigate today's shifting learning environments.

## 2 PLANNING THE ONLINE COURSE—THE BIG PICTURE

Well‐designed online courses should consider two distinct aspects: the course content and the workflow for learning. As scientists, we naturally default to trying to share with our classes everything we know and love about our research field. With the shift online, now is the time to hold back: Less is more. Though paring back will result in covering less content than you normally would with an in‐person course, the trade‐off is that the content will better align with learning goals for the course (Brewer & Smith, 2011).

Organize the content around 3 or 4 big questions that capture the overarching learning goals you want students to take away from the course. The big essential questions help prioritize what content is the most important for students to know in order to gain understanding. Backward design principles may help a course designer identify the big ideas and important enduring understandings that students should retain (Wiggens & McTighe, 1998; for quick reference, http://udlguidelines.cast.org/). When done well, essential questions serve to align content with learning goals and make clear to students the “why” of learning, and not just the “what.” Once students know why they are learning something, they are more likely to feel motivated and invest time in the learning—even when the material is difficult.

In NT’s upper‐level *Developmental Biology* course, the three essential understandings (shared with students on the syllabus) were as follows: (a) How do cells communicate to form an embryo? (b) What processes control cell and tissue patterns? and (c) How do cells organize into functional structures? In JC’s *Introductory Biology* class, the three essential understandings were as follows: (a) How is biological information stored and passed on? (b) How do organisms evolve? and (c) How do organisms interact with each other and their environment? Outlining your course with essential questions like these is invaluable when developing course activities and assessments such as examination questions (see *Assessments*).

### 2.1 Code of conduct

Just as in a physical classroom, where you expect students to arrive on time and not talk during class, you should also expect students to have a standard and expectation of behavior while in a live online class. Don't be afraid to also set a “code of conduct” for behavior when online (Tables 1 and 2). In fact, you may want to spend a few minutes during the first synchronous session of the term having students come up with their own code of conduct and behavior. Students are self‐aware and easily identify their own challenges and behaviors. After students come up with their code, feel free to make suggestions. While students in *Developmental Biology* created an effective code of conduct (Table 2), given the experience of this past term NT would add, *Will not attend class while lying in bed!* Tell the students why you want to make the addition and then ask the class if they agree with your suggestion. It is important to get buy‐in from students on what is acceptable behavior while online and what is not. Post the agreed‐upon code on the course LMS or add it to the syllabus so everyone has a copy of it. The code can be used by the instructor and students to call out student behavior during the term. One of the rules written by students was *Stay off the phone during live class time* (Table 2). During a class early in the term, two students were clearly texting and laughing during a discussion. A quick reminder in the Zoom chat box about the code of conduct put a stop to it.

**Table 1:** Quick tips for synchronous live online classes

Schedule synchronous class time at the same time every week. Use the same Zoom or Google Meet's link to increase reliability and decrease confusion. Record all synchronous classes and make immediately available to students in a single location. Mix up the activities—the instructor should not talk the entire time. Use breakout rooms periodically during class to engage students in discussions about content. Have students develop their own code of conduct and behavior while in live classes.

**Table 2:** Code of conduct devised by *Developmental Biology* students on the first day of class

**Common rules that we will abide by…** Discussions and chatting will be focused on class material and be respectful of one another When someone is talking or sharing, everyone agrees to respect what they are saying and the space/time in which they are speaking Stay off the phone during live class time Stay in a place where there is not a lot of background activity Try to keep facial expressions supportive, even if you do not agree with someone Maintain honor code with respect to online resources Mutual willingness to engage

### 2.2 *Synchronous* versus*asynchronous*

**Figure 1:**
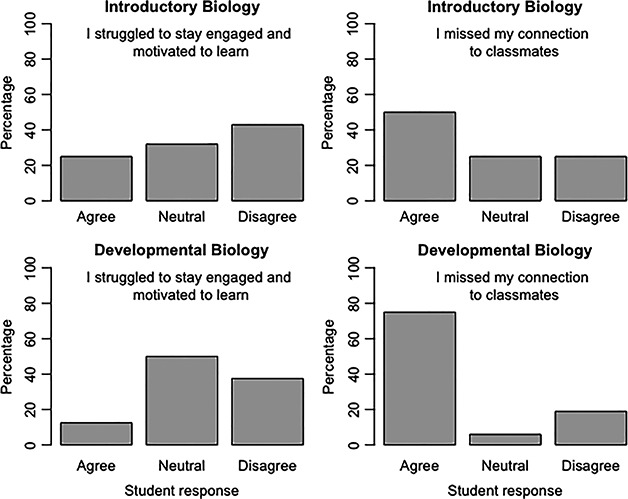
Student responses to two questions regarding their engagement, motivation, and connection to classmates in spring 2020 *Introductory Biology* and *Developmental Biology* courses. Percentage responses to each question are derived from 65% (28/43) of *Introductory Biology* students and 67% (16/24) of *Developmental Biology* students who completed the course

When weighing how much of the course should be synchronous (instructor and students online at the same time) versus asynchronous (content that is provided for students to do at their own pace), consider several questions: (a) What is the purpose of, and value‐added to, the content in each mode? (b) Is the schedule and balance of activities predictable and consistent on a week‐by‐week basis? and (c) How can the course design maximize flexibility and student engagement? If you are thinking of delivering all course content “live,” be aware that students may be in dramatically different time zones and you may be asking some of your students for their attention at odd hours of the night and early morning. Even students in the same time zone may be competing for Internet bandwidth, computer access, or quiet space with family members. A department colleague that had several students in Asia offered an additional weekly class to better accommodate the time difference, but recording all synchronous sessions and making them readily available for later viewing can be enough for students to keep abreast of the class and stay connected (Figure 1).

**Figure 2:**
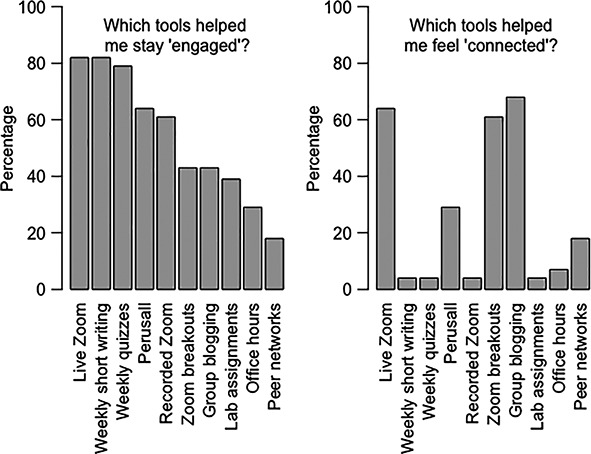
*Introductory Biology* students’ responses to questions related to specific tools that fostered engagement and connectedness

Synchronous activities are an opportunity for students to connect with each other and engage with the content in a dynamic way. In survey responses, students reported that synchronous live sessions were the weekly activity that most helped them feel engaged with the course (89% and 93% for *Introductory Biology* and *Developmental Biology*, respectively) and connected (64% and 81%, respectively). Synchronous activities can be fun and active ways of reviewing content learned asynchronously to deepen understanding. Consider using case studies to promote application of the asynchronous course content and use real‐world and recent topics in the news to provoke discussion and demonstrate immediate relevance. Breakout rooms are effective at giving students a virtual space to work on problems together and then report back to the group (Table 1). Conversations in breakout rooms were not proctored or moderated. Instead, students were given Google Docs to fill out together, and progress was monitored in real time by the instructor. Interestingly, students ranked breakout rooms high for connection and low for engagement (61% and 43%, respectively), suggesting a perception that breakout rooms served mostly a social purpose (Figure 2). Assessment of the Google Docs, however, indicated that students were fully engaged with the content during the breakout sessions as they completed assignments collaboratively.

Not all teaching and learning needs to be done in synchronous class meetings, a lot of student learning can be asynchronous (Degges‐White, 2020). Indeed, we argue that asynchronous activities can be the backbone for student learning. Asynchronous activities allow students greater flexibility to schedule around other courses, and obligations to family and employers, and accommodate their different time zones. Another advantage is that students can work at their own pace. Asynchronous content can include reading assignments, group work, and prerecorded lectures. A number of tutorials and professional videos are available online either through the textbook publishers or on YouTube and Vimeo. Before dedicating hours to recording and editing your own videos, you may find existing content easier to curate. Many scientists responsible for important discoveries and concepts have publicly recorded lectures, and hearing directly from the scientist can be effective for student learning and engagement (see Section 4.1). Keep your own videos short (~20 min) to better hold student attention when viewing. To make sure students are watching video lectures and getting the information they need out of them, quiz questions can be embedded within videos or postvideo quizzes can be used to reinforce information. Be careful not to overburden students with too much content—if you normally have 4 hr of lectures per week in person, realize that not all of your time in an in‐person class is spent on content delivery, and prerecorded video time should be cut to at least half.

### 2.3 Assessments

Just as ways of delivering information should be reworked for online teaching and learning, assessments and examinations also deserve fresh rethinking. Faculty are often concerned about the integrity of student work: Will students be more likely to use prohibited online resources? Collaborate/cheat on examination questions? Submit original work? When these questions are the concern, the administration of live examinations may be necessary. Examinations can be proctored live via Zoom/Google Meet classes or using proctoring software and services (such as ExamMonitor, Proctorio, Proctortrack, and ProctorU). Such tools videorecord students taking examinations; videos are stored until they can be screened by the instructor. While such tools may be convenient for the instructor, these proctoring services have issues regarding data security including where the videos are stored, who has access to them, and for how long a period of time (Dimeo, 2017). Further, students report that the video recordings during examinations felt intrusive, and sends a message that the student is not trusted (Dimeo, 2017).

Live proctoring may not be necessary where students are assessed on key concepts. A course that is constructed around essential concepts or understandings (see *Planning the online course—The big picture*) can frame assessments away from questions that test memorization to original‐thinking questions that evaluate those concepts and understandings. As a result, examinations and other assignments are less prone to cheating or plagiarism. The final examination for *Developmental Biology* asked students to answer each of the essential understandings using examples from the course in an open book and notes format. Examinations in *Introductory Biology* were also open book and open notes, and focused on key concepts rather than first‐ or second‐order Bloom's taxonomy, content‐focused questions. For example, multiple‐choice questions queried the mode of evolution—natural selection, genetic drift, or gene flow—based on a set of facts, rather than seeking definitions that can be looked up on Google.

**Figure 3:**
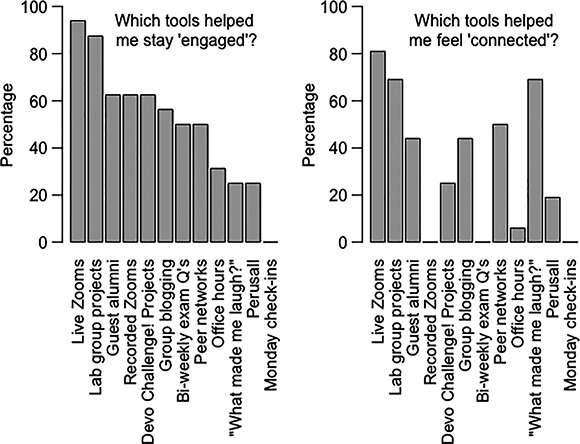
*Developmental Biology* students’ responses to questions related to specific tools that fostered engagement and connectedness

To maintain motivation and engagement, examinations can be shifted from longer midterm‐style examinations to more frequent, shorter, and self‐scheduled assessments. Our students reported that frequent assessments were among the most motivating tools in our classes (Panel 1, Figures 2 and 3). An advantage to administering examinations as lower‐stakes events is that, if a student should do poorly on one examination because of health, family, or connectivity issues, then their performance has a lower impact on the total course grade compared with the larger impact had of a few big examinations.

One form of shorter assessments is to post one examination question ahead of time, for example, at the beginning of a 2‐week unit, and allow students to work on the question at their own pace until a set due date several days after the unit content has ended. Questions can be written as fictional scenarios that promote student thinking and application of concepts that cannot be directly looked up. For example, for a unit of limb development and identity, *Developmental Biology* students were asked to come up with a model for how a hippogriff, a fictional animal in the Harry Potter series, developed four legs and two wings from an ancestral horse. The question required an understanding of limb field initiation and pattern formation, and how evolution modifies the body plan. Five similar examination questions in *Developmental Biology* were provided over a 10‐week term for a total of 20% of the final grade in the course (4% each). This format gives students a predictable expectation and flexibility of when they can work on the assignment and empowers students to be independent.

In larger classes, frequent, shorter, multiple‐choice quizzes are an effective way to provide students a “check‐in” to gauge their understanding of the material and may cover smaller course subunits than major examinations. In *Introductory Biology*, six quizzes were administered over the 10‐week term for a total of 12% of the final grade; they were administered and automatically graded using LMS. Such quizzes can be cumulative, encouraging retention of material through the course (Brown et al., 2014). Note that frequent quizzes and “ahead of time” examination questions can be used in combination.

Examinations and quizzes are not the only avenues for assessing student learning. In *Developmental Biology*, examinations represented only 20% of the final course grade, while annotated readings in Perusall (10%), reflective blog entries with peer feedback (20%), a NSF grant proposal (30%), and an individual final project (20%) made up the remainder of the course. Group work and collaborative learning were assessed differently depending on the intended learning outcome of the assignment (see discussion in *Assessing group work*). In *Introductory Biology,* examinations and quizzes were worth half of the final course grade. Perusall readings (15%), laboratory assignments (15%), in‐class participation (10%), and reflective writing assignments including group blogs (10%) made up the rest.

## 3 Peer Groups

Collaborative (group) learning is a well‐established tool that promotes student learning (Loes & Pascarella, 2017; O'Donnell & O'Kelly, 1994; Springer et al., 1999). The effectiveness of collaborative learning is built off the work of the social constructivist Vygotsky who believed that social interaction is essential for cognitive development (Vygotsky, 1978). Group work increases student engagement, leading not only to higher achievement and deeper learning, but also a sense of belonging. During the widespread COVID‐related shifts to online learning, our students reported that they missed peer interactions inside and outside the classroom (Figure 1). Thus, the use of groups has the potential to improve students’ emotional well‐being by providing social connections that otherwise might be frayed (So & Brush, 2008).

Though most of the empirical study and practice of cooperative learning are based on face‐to‐face, in‐person interactions, the benefits transfer well to online learning (Curtis & Lawson, 2001; Gokhale, 1995; Jeong & Hmelo‐Silver, 2016). Most of the collaborative assignments we utilized in spring 2020 were modified versions of ones that we have routinely assigned during on‐campus instruction. Students can interact in groups through laboratory exercises, collaborative examination questions, shared reflective writing assignments, or case studies/problem‐solving. Groups can be engaged in either long‐term projects over multiple weeks or short 15‐min problem‐solving in breakout sessions during a synchronous class meeting.

A variety of different strategies can be used depending on the learning goals. Online breakout rooms can be used for 15‐min problem‐solving activities or case studies discussions (Table 1); Zoom and Google Meet can randomly assign groups so that students can discuss problems and replicate the in‐class huddle. Google Docs can facilitate real‐time student collaboration and permit the instructor to remotely monitor a group's progress. For long‐term projects, assign roles to students within a group, defining those roles clearly for students and assessing their performance. In *Developmental Biology*, students worked in groups on a backyard science project and with the data collected, collaborated to write NSF‐style grant proposals (Cole et al., 2013). Within each group, students were assigned roles to play: laboratory technician, graduate student, postdoc, grant coordinate, and grant editor. After the proposals were submitted, students ran “study sections” to review and score the proposals. As part of the project setup, students were told that effective teamwork was an essential but difficult skill to develop, and one that would serve them well in future careers. If during group work students complain about their group members, ask them what they have done to correct the behavior and talk to them about the importance of troubleshooting group dynamics. Avoid trying to “fix” the problem for them. When a long‐term project is completed, ask students to reflect on their performance in the group, and provide feedback on how the group members worked together. Having the group work assessed and clearly articulated in the syllabus emphasizes the value you place on effective group work.

### 3.1 Reflective writing and peer feedback

**Figure 4:**
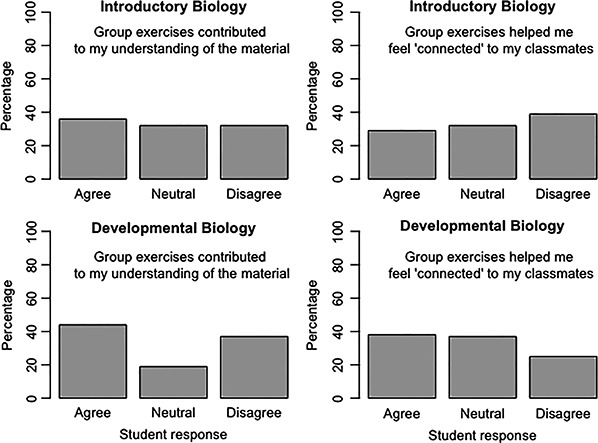
Student responses to two questions regarding how group exercises contributed to their understanding of course material and connection to classmates

When students have the opportunity to reflect on their learning process without the fear of being wrong, they can improve their critical thinking skills (Ambrose et al., 2010). Providing a low‐stakes way for students to evaluate evidence and synthesize concepts is the main purpose of reflective writing. Tying the content of the class with current news, ethics or politics is a great way to engage and promote intrinsic motivation. Combining reflective writing with peer feedback has the added benefit of extrinsically motivating students to take the assignment seriously. Peer feedback provided a platform for students to interact and exchange ideas about the course material: gaining deeper understanding and realizing new connections with past knowledge that they had not considered on their own (Table S1). Though student survey responses indicated an even split between students who agreed versus disagreed that the group reflective writing assignments helped them stay motivated or feel connected to classmates (Figure 4), reflective writing was among the most often chosen tools that helped engagement and connection (Figures 2 and 3). Many students also noted that the group blogs were effective in creating connections with their peers (Table 4).

**Figure 5:**
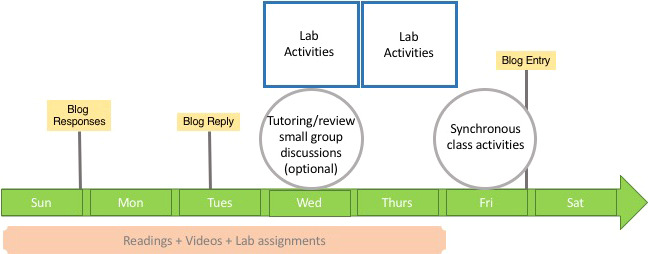
A visual representation of the weekly activities schedule for *Developmental Biology*, reproduced from NT’s syllabus. The use of graphics in syllabi improves accessibility and simplifies expectations. A student can easily glance at this image and know that they have one required class at the end of the week, a mid‐week optional tutorial and a laboratory (the course had two laboratory sections, and students were enrolled in either the Wednesday or Thursday section). Writing reflections (blogs) were due on Fridays by midnight, and responses and replies were due Sundays and Tuesdays, respectively

In JC’s *Introductory Biology* class, reflective writing took the form of group “blogs" in which groups of 5–6 students answered weekly questions and commented on each other's answers. Questions were posed to each group at the beginning of each week via LMS, and students were expected to post their answers by the end of the week, and comment on their groupmates’ posts by the beginning of the next week (i.e., Figure 5; Table S1). Questions were open‐ended, giving students the opportunity to express their opinions or interests. Early in the term, when students did not know their group members well, questions were designed to help develop familiarity and to “break the ice” (Table 3). Subsequent questions were built off course topics, but importantly they were not model “test questions.” For example, students were asked to describe how artificial selection was applied to a wild animal or a nondescript dog/cat, etc., to give rise to the traits of a domesticated animal or pet breed of their own choosing (Table 3). This post allowed students to apply the mechanism of artificial selection—and, by extension, natural selection—to a specific case that they find interesting. Another post let students choose and describe their favorite ecosystem and its key ecological factors. The group blog assignment culminated in a reflection of how their writings developed over the term, both in terms of how they approached biological topics but also their interactions with their groupmates.

**Table 3:** Examples of topics in blogs and reflective writing assignments

**Introductory Biology** “Share with your groupmates where you are. How are you spending your time? Next, why are you taking this course? What is your favorite aspect of biology?” “Some cancers run in families – that is, certain families have higher susceptibility to particular forms of the disease than others. Using your knowledge of gene inheritance and how information in genes turns into cell function, explain why this is the case” "Pick a domesticated animal – or a particular breed of dog, cat, etc. – and describe the process of artificial selection beginning with a wild animal or a relatively unremarkable type of dog/cat/etc. Finish your description by drawing a parallel between artificial and natural selection” "What is your favorite kind of natural ecosystem? Using your book's details about biomes and your own research, what are the ecological features in this habitat – the kinds of plants and animals, the likely abiotic factors that make it look like it does” **Developmental Biology** “Read a JAMA article on parental preconception exposure to phthalates (Zhang et al., 2020) and the corresponding NYTimes summary of the same study (Bakalar, 2020). What new understanding about environmental toxins is revealed in this study? What questions are left unanswered by this study ‐ what are your 'burning' questions after reading the article? How well did the NYTimes summary portray the findings of this study? If you could edit the NYTimes summary, is there something you think should be included/added?” “Earlier in the term you learned that cleavage of the blastomeres happens differently in different animals depending on the amount and distribution of yolk in the egg (see individual organism cleavage videos for a refresher). This week you learned about the cell movements and cell signaling that specify the germ layers during gastrulation. Below are videos depicting gastrulation from 5 different organisms. Watch these videos closely and answer the following questions: What are all the things you see? What does this make you think about? What does it make you wonder? Remember this is reflective work, if you are answering any one of these questions in 5 min you aren't thinking deeply” “So far this term we have addressed 3 important concepts when talking about ‘patterning the body plan’: gene expression, fate maps and gene mutations. We've also learned that patterning involves cells communicating to each other through signals. While patterning of the body plan is taking place, cells move through time and space to give rise to those 3 basic germ layers. The germ layers ultimately give us all our tissues and organs. For this week, please reflect on the relationship of cell communication with cell movements and how we build the 3 germ layers. Perhaps consider an area of confusion and try to delve into it, or consider something that you found surprising about the way cells move through space and continue to communicate with each other. Why was it surprising (or confusing), what did you do to uncover or understand it better? How did this change the way you think about cells or even more broadly about biological processes?”

Weekly reflective writing in *Developmental Biology* had higher expectations, as one would expect in an elective class for biology and biochemistry majors. Questions gave students the opportunity to critique primary scientific literature, learn new material, or form their own connections between course topics (Table 3). Students were organized into randomly selected groups of 4, and groups were switched every other week. Within a group, each student posted their response to the weekly prompt on the LMS forum and provided feedback to every post in their group (Figure 5). In order to facilitate constructive and meaningful feedback, students had to choose one response that they received to reply to, thus stimulating a dialogue about the course material (e.g., see Table S1).

### 3.2 Assessing group work

The strength of peer group work is that students are encouraged to rely on each other to learn and understand the material, building independence and self‐motivation. Often, faculty grade for “correctness,” which can limit deep thinking as students cater to what they perceive to be a single “right” answer. The intention of the reflective writing blogs was different: to pose questions with complex answers and to encourage students to take risks with their ideas and thinking by being *incorrect* and not penalizing for mistakes. Thus, the weekly posts and peer responses were not read, responded to, or evaluated on a weekly basis by faculty. Instead, students were given the room to experiment with and exchange ideas. We did occasionally check‐in to monitor the groups and make sure students were continuing to submit the blog posts and respond to peer entries. Twice during the term during *Developmental Biology*, and once during *Introductory Biology,* students wrote and submitted a self‐assessment of their performance, gauging their own growth as a learner. The self‐assessments, content quality of blog entries, collaboration with peers, and timeliness of entries and feedback were scored at the mid and end of term. Thus, the assessment of reflective writing entries and peer feedback was focused on student contribution and participation rather than on correct content.

The approach to assessing the NSF grant proposal in *Developmental Biology* was different. Students worked in groups of 6 and collaborated on five written assignments (Methods & Results, Grant Outline, NSF Grant Proposal, NSF Peer Review, and NSF Scoring sheet) that were submitted and graded. Students within a group received the same grade for these written assignments unless there was a significant discrepancy in the contribution of a student. In addition, students within a group were asked to assess each other and write an evaluation of how the group worked together at the end of the project. This assessment tool was used to generate an overall collaboration grade for each student and was worth 20% of the NSF grant proposal project grade. The NSF grant proposal was worth 30% of the course grade; thus, the collaboration grade became a significant factor in personalizing the final grade of this assignment.

From these examples, it is clear that group work can serve different purposes, promoting collaboration, supporting risk‐taking, and/or instilling resilience. Assessing group work should reflect the intended learning outcome or skill gained by the activity as opposed to the more common content‐driven approach used to grade.

## 4 Outreach to Broader Scientific Community

When instruction is virtual and students’ worldview is greatly reduced, hearing voices beyond students’ small network is more important than ever. Bringing the broader scientific community into the online classroom allows students to hear experts in their own voices, which is a powerful way of humanizing scientists. The broader scientific community can be brought into the classroom in several ways: as experts in their research fields or as young scientists finding their career paths. Selecting expert voices that reflect the diversity of your students can have an added benefit of helping students make a personal connection with scientists and help them realize that being a scientist is an attainable goal.

### 4.1 Bring in the expert—inviting scientists into the online class

Guest research seminars are a staple of college and university science departments, but conversations with scientists can be a powerful tool in undergraduate classes, too. The conversation differs from the more traditional campus “seminar,” as we do not ask the guest to deliver prepared slides. Because students have prepared by reading the paper beforehand, the guest can assume a level of shared understanding of the topic. Furthermore, the absence of faculty (aside from the instructor) gives students the permission to engage in a way that is usually absent from more open, auditorium‐style seminar presentations. The result is usually an intimate, dynamic, and energetic session.

With proper scaffolding, even relatively inexperienced undergraduates can carry on a high‐level conversation with experts. Consider what assignments are going to be given ahead of time. Are students going to read and discuss the guest's most remarkable or current research paper(s)? Will background on the guest's personal history be given? Perhaps there is a seminar by the scientist available online for students to watch and write a reflective piece beforehand.

Guest scientists can be invited to participate via Zoom, Google Meet, or Skype. The format of the meeting can be a Q&A session, moderated by the course instructor. Students may read and annotate a paper by the guest, and generate their own questions. We recommend the questions be submitted in advance, so that the instructor can organize the questions by topic and manage the flow of the discussion. Students' questions may be forwarded to the scientist ahead of time, so that the guest has the opportunity to reflect on the questions before the live class. Questions about the paper can range from motivation for the study to methodological details to implications. But questions can also touch on the scientist's own background and career path, thus humanizing the stories behind the published paper.

Besides adding to the voices that students hear from and making students feel connected to the broader scientific community, such conversations are also valuable for reinforcing the nature of science as a *process*. Students can hear “how the sausage was made”—details that are usually sanitized for publication. One guest to a class we taught described a serendipitous observation that led to a paper in the journal *Nature*—a moment of humility and accessibility that students rarely see in published science. They can also hear stories of personal motivation, challenge, or inspiration that they otherwise would not imagine. Finally, guests can be chosen to highlight diversity in science and encourage members of underrepresented groups to see themselves belonging in science (Gewin, 2020).

### 4.2 Bring in recent alumni

In addition to the challenges of online learning, the pandemic brought a frozen and uncertain job market for graduating seniors. The class of 2020 had to navigate finding jobs while being separated from their network of friends, professors, and institutional resources that normally support them through this process. The class of 2021 and beyond will also face unknown obstacles as they chart their futures. The presence of recent alumni provides a tangible link for students to imagine life after college.

When inviting former students who graduated within the last few years to join your class, consider selecting students of both diverse backgrounds and diverse career interests. These could include professionals pursuing careers in health professions, veterinary medicine, industry, graduate school, and clinical and basic science research. This is an opportunity to highlight paths besides the academic or premed tracks that can dominate career discussions. Some guests may admit to not having a clear career focus of what they wanted to do when in college.

Before the online class meeting, ask alumni guests to share what they are currently doing and their path from undergraduate to their current positions, and in addition, provide several "prompts" ahead of time to facilitate the conversation when online, such as:

- *What did college prepare you well for?*
- *I wish someone had told me _______ when I was an undergraduate*.
- *After being out of college for a few years, I now realize/know_______*.
- *Words of advice: ______*.

In the exchanges with alumni, current students learned the importance of networking and the exposure to different experiences and career options. As alumni identified the skills they learned in college that helped them the most in their first jobs, current students were able to reflect on their own existing experiences and personal learning goals. They also heard reassurance that it is okay to take the time needed to explore career options. Students reported that meeting and hearing about the alumni experiences were helpful and reassuring. In discussions with alumni guests, students opened up about different pressures they felt either self‐imposed, from parents and families, and/or from peers about what their careers “should be” and how they struggled to reconcile these pressures while trying to stay open to new options. One student reported “I think everyone is a bit worried about life after college, especially at a time like this, so it is reassuring to hear alumni speak about what they're up to.”

We have found that the guests find the experience rewarding, as well. Recent alums are eager to connect with current students and their former professors, and an invitation from their undergraduate institution makes them feel connected and valued. This was evident in our invitees’ openness to share their experiences and reflecting on their own career paths. Alumni welcomed current students to reach out to them and offered them help in finding positions or for general advice.

## 5 High Touchpoints

The spring 2020 disruptions extended beyond the ways that our courses were delivered and managed. Students and instructors, alike, were isolated and had to navigate drastically different learning and working environments compared with ordinary circumstances. Depression, anxiety, and loneliness hit record highs among college students this spring (Koetsier, 2020). We found that, while students benefited from a greater emphasis on process and scheduling, everyone needed increased flexibility in assignments and a more personalized experience. Frequent, personal, check‐ins with individuals or with smaller groups garnered praise from students (Table 4). Online instruction requires more work to keep track of students that fall behind. It can be more difficult to identify those who need help—academically or emotionally—when content is delivered fully or partially asynchronously. Student participation in the course was monitored not through their attendance of synchronous classes, but rather through their contributions to weekly blogs and group activities. In this way, students not taking part in assignments were identified and contacted by email. We both used spreadsheets to keep track of email check‐ins and correspondences, including dates and student responses in an effort to make sure that students didn't “disappear.”

**Table 4:** Selected student responses to survey

**Planning** The consistent Zoom sessions and Perusall assignments helped me stay engaged and motivated to learn the material It was hard to stay engaged and motivated but the assignments (blog posts, Perusall, Friday Qs, quizzes) helped me a lot Having a weekly list of assignments and events was SO SO SO helpful I think the organization of the [LMS platform] and having everything in one location was very helpful for online learning in this course. I also liked the pairing of videos and textbook reading as they complement one another well I really enjoyed the class and liked how we focused on broader topics Having due dates and assignments that tested our knowledge helped me stay engaged, because otherwise I wouldn't feel the motivation to understand it as well **Peer groups** I think that online learning could feel a bit hopeless and pointless at times, however group work and weekly reflections help to ground what we had actually learned I liked the blog posts and Friday Qs because they help guide and center the important topics learned throughout the week The Perusall readings and having to leave comments made me think about the material more critically because I got to see other peoples questions and then think about possible answers. I liked the blogs because it did connect me with my classmates The more online activities and interactions with my classmates made this class really productive for me to learn [The blogs made me] put forth the effort to test myself and learn about this topic that was previously giving me trouble. By doing so, I surprised myself with the end product. I did not know I was capable of learning something in such a fashion, and this method of understanding gave me a much more in‐depth learning than what I would get through simply reading notes. This was a more effective method of studying which I had not previously been aware of I think group collaborations was the best way of trying to stay connected to classmates because it encouraged people to have conversations outside of class **High Touchpoints** It was definitely hard to stay motivated at times but I think little things like the "what made you laugh this week" segment and weekly guests kept everyone engaged and made school feel a little more fun and relieved some of the pressure and stress You were incredibly straightforward in what you expected from us and had a lot of compassion during these unsettling times Email reminders really helped with organization and assignments this term which was helpful without in‐person meetings I think that this was my favorite class this term as you were able to incorporate many different activities into the term that kept me interested and motivated I really enjoyed your teaching style.. incredibly straightforward in what you expected from us and had a lot of compassion during these unsettling times. It was very clear that you cared about our learning and I think every assignment that we did reflected this

A clear and predictable schedule is imperative for reducing stress in students and promoting engagement. It helps reduce student confusion about when things are due, and students less often hand in assignments late or fall behind. A survey that we each distributed early in the term (week 3 of the 10‐week term) found that students were overwhelmed by the number and range of online tasks—multiplied by the number of classes students were taking. In response to this feedback, we made adjustments to the structure of our courses, including posted weekly Google Doc or LMS schedules of topics and assignments, and reviewed “agendas” for each live Zoom meeting class that more closely resembled business meetings than class materials. NT devoted a few minutes each week to a live Zoom session whose sole purpose was to provide an overview and context for the upcoming week's topics and assignments. When assignments were clearly laid out and communicated regularly, we received few requests to change deadlines.

Synchronous tutorials should be set at consistent times, for example, on Wednesdays with case study activities on Fridays; regular assignments, such as writing reflections or blog posts, should be due the same day each week (Figure 5). When scheduling weekly synchronous class time, use the same Zoom or Google Meet link for all the classes so students can easily set their schedules and readily find links for the face‐to‐face time they look forward to (Table 1). Make all recorded content available in a single location. Students were very appreciative that their input on course structure was sought early in the term and that we had implemented changes in response to their feedback. The measures we implemented in response to student feedback was successful as our end of term student surveys revealed that such consistent and clearly communicated schedules were valued by students (Table 4).

In addition to having a clear schedule, find alternative avenues for students to reach you, pose questions, and seek out additional help. Speaking up during live Zoom sessions—especially in large classes—may be more difficult than in an in‐person format, and some student populations may find faculty office hours via Zoom intimidating (this is reflected in the low ranking of office hours for engagement and connection, Figures 2 and 3). Opportunities for students to ask questions can be either structured or take a more informal approach.

Each week, *Introductory Biology* students answered two “Friday Q’s” via LMS—one of which was a check‐your‐knowledge of the week's material, and the second was an open‐ended prompt: “What are your lingering questions about this week's materials?” The answers could be answered individually or collated and used to guide follow‐up presentations. The Friday Q’s were among the most cited tools to maintain student engagement in surveys (Figure 2). In addition to Wednesday live tutorials for explaining complex problems in *Developmental Biology*, an “ask the professor” forum was created on the LMS for students to post questions and for the entire class to see the instructor's responses. In this way, everyone got to see each other's questions and it also reduced students repeating the same question, decreasing the number of emails required by the instructor.

For informal “run‐ins” with students, arrive 10 min early and linger at the end of your synchronous online class time. We found that students who came early or did not immediately leave at the end of class often had something on their minds. A simple “How is it going?” may help a student gather courage to share either questions about the material, or personal issues preventing the student from focusing or completing assignments. We also acknowledged broader social topics including the global pandemic and #BlackLivesMatter. Occasional humor can also lighten the mood in an effective way. NT invited her *Developmental Biology* class to share “What made me laugh/what brought me joy” experiences once per week, where students took turns sharing a light moment from their week and “tagging” peers for the next week's sharing. JC solicited “Dad jokes” from his *Introductory Biology* class and then rolled “good” ones out on a regular basis. Find something you are comfortable doing and that humanizes you and the situation we find ourselves in. A little empathy goes a long way.

## 6 CONCLUSION

As Union College's president likes to say, “The virus gets a vote.” There is no way of knowing how long or how often faculty will be required to teach online. What is clear is that the need to deliver high‐quality online learning to students is here for the unforeseeable future. We were fortunate to be at an institution that is on a trimester system, so our spring term was offered fully online. This afforded us the opportunity to fully engage with the online platform including trying out and adapting tools we normally use in‐person. What we learned is that a well‐curated online environment can be engaging when the focus is shifted to student‐centered learning. A course design around big questions that emphasizes group work and empowers students to make choices can be motivating for students. By switching the burden of learning to students, students gained ownership and resilience throughout the term. Outreach to the broader community of research scientists or early career alumni networks helps demystify science and enrich the learning experience. Finally, we encourage all teacher–scholars to be socially present for students, set a tone that promotes belonging, send students weekly announcements or personal “pokes” when they seem disengaged. Ask students for feedback and listen to them—we have much to learn from each other.

## Conflict of interests

The authors have no competing interests to declare.

## AUTHOR CONTRIBUTION

**Nicole Alexandra Theodosiou:** Conceptualization (lead); Data curation (equal); Formal analysis (equal); Writing‐original draft (equal); Writing‐review & editing (equal). **Jeff Corbin:** Data curation (equal); Formal analysis (equal); Writing‐original draft (equal); Writing‐review & editing (equal).

## Supporting information

Table S1

## Data Availability

All data are available in the manuscript text.

## Appendix 1

### PANEL 1—SURVEY OF STUDENT EXPERIENCES IN TWO UNDERGRADUATE COURSES IN SPRING 2020

We surveyed students in our two courses about their experiences and how various elements of the courses contributed to their motivation to learn and their feeling of connection to the rest of the class. Surveys were distributed at the end of the term via email, and anonymous responses were collected via Google Form; participation was optional and was granted an exemption from review by the Union College Human Subjects Review Committee as per 45 CFR 46.104(d)(2).

Twenty‐eight of 43 (65%) *Introductory Biology* students and 16 of 24 (67%) *Developmental Biology* students completed the surveys. Some students in both classes agreed with the statement that they “struggled to stay engaged and motivated to learn,” but this was a minority view (Figure 1). There was more agreement that they “missed their [normal] connections” to classmates (Figure 1).

Among the tools that *Introductory Biology* students reported as helping stay engaged with class material were twice‐per‐week live Zoom sessions, Friday Qs (see *High Touchpoints*), weekly quizzes, and Perusall collaborative textbook reading assignments (Figure 2). The most often cited tools that students reported as helping to stay connected with classmates were group reflective writing assignments (see *Peer groups*), live Zoom sessions, Zoom breakout rooms, and Perusall assignments. For comparison, students’ own peer networks ranked relatively low compared with the above tools, and recorded Zoom sessions were only cited by only one student as helping maintain connections (Figure 2). In *Developmental Biology,* the tools most often cited as effective in keeping students engaged and motivated were live Zooms, group laboratory assignments, Zoom conversations with guest alumni (see *Outreach to broader scientific community*), a final project the “Devo Challenge!”, and group reflective writing assignments (see *Peer groups*) (Figure 3). Students reported that such tools as live Zoom classes, group laboratory assignments, and sharing “What made me laugh” stories (see *High Touchpoints*) helped them to stay connected with classmates. Peer networks were more helpful in maintaining connections for *Developmental Biology* students than it was for *Introductory Biology* students, as they were cited by 50% of the respondents (Figure 3).

Participation in the peer group reflective writing assignments was high in both classes (89% and 81% of *Introductory Biology* and *Developmental Biology* students, respectively, contributed each week). Over 60% in both classes reported that writing the posts and integrating course material became easier as the term wore on, as did providing peer feedback (Figure 4). Among *Introductory Biology* students, 36% reported that the group reflective writing blogs helped them gain understanding of the material; 29% reported that the blogs helped them form connections with peers. *Developmental Biology* students responded in a similar fashion: 44% of the students reported that the blog assignments contributed to their understanding of the course material, while 38% said they did not. 38% of the students reported that they helped foster connections with peers, while 25% said they did not (Figure 4).
