# Supplementary material for: Redesign your in‐person course for online: Creating connections and promoting engagement for better learning
Source: Ecol Evol. 2020 Sep 29;10(22):12561–72. doi: 10.1002/ece3.6844 (PMC7679538; doi:10.1002/ece3.6844)
Supplement: Supplementary file 1 — Table S1 [file ECE3-10-12561-s001.docx]

**Supplemental Table 1: Samples of reflective writing and peer feedback**

**Sample blog prompt from *Developmental Biology*:**

So far this term we have addressed 3 important concepts: gene expression, fate maps and gene mutations. We've also learned that patterning involves cells communicating to each other through signals.  While patterning of the body plan is taking place, cells move through time and space to give rise to those 3 basic germ layers.  The germ layers ultimately give us all our tissues and organs.

For this week, please reflect on the relationship of cell communication with cell movements and how we build the 3 germ layers.  Perhaps consider an area of confusion and try to delve into it, or consider something that you found surprising about the way cells move through space and continue to communicate with each other. Why was it surprising (or confusing), what did you do to uncover or understand it better? How did this change the way you think about cells or even more broadly about biological processes?

**Student A reflection entry:**

Over the course of learning about the different processes that allow for development, I have continually been impressed by the intricate signaling and movement that cells undergo. Particularly, the fact that every area of the developing embryo seems to have a specific gradient or mechanism that makes it unique. All of this comes together in allowing the  different cells that make up our body to form our tissues and organs from the 3 germ layers. One area that I had trouble understanding was that of hinge points and apical constriction. The idea that the sheet of epithelial cells begins to bend in 2 specified locations seemed confusing: how do they know where to bend? What allows them to do so? How does a sheet become a 3D structure? I studied the diagram of these hinge points and was able to see the specific changes that occurred to the cells and at what locations. I then read that the cells at the MHP and DLHPs adopt a wedge-shape morphology along the apicobasal axis, but that still left me with questions: what even is the apicobasal axis? I was able to gather that it is a top to bottom type axis, which made sense when looking at the diagrams of how the cells are wider basally than apically. I further learned that this is caused by localized contraction of actinomyosin complexes at the apical border and is known as apical constriction. I still wonder about whether a gradient exists for the actinomyosin complexes such that it favors the contracting of the apical side? Another factor that allows for the thickening of the basal side is retention of the nucleus, utilizing the fact that apical constriction can pair with the basal location of nuclei to promote these wedge shaped cells. Lastly, I am starting to understand the physical mechanism of how this can happen, but why does it happen? Looking into it more, I am not surprised to find out that we have BMP to thank for these hinge point formations. In this case, BMP inhibits MHP and DLHP formation whereby repressing BMP with noggin allows the hinges to form. Additionally, I was not aware until lecture today of the role of N-Cadherin and Shh! This seems to be influential in initiating the MHP, but does it have any influence in the DLHP formation? Diving into this concept definitely allowed me to understand how hinge points arise and further see the influence they have on shaping the developing embryo. Understanding this process has emphasized the role of controlled signaling of other molecules. The biggest take away from this was how repression of molecules can be just as influential as expression. I am still curious as to how many cells are usually encompassed in the MHP and DLHP regions. Would love some insight if anyone has any!

**Student B response to student A:**

I really liked how clearly you laid out your thinking in this post! The way you explained how your understanding increased in small increments and the questions you had after each new understanding made it really easy to follow and I think reflects the way a lot of us probably think/feel! I can definitely connect to finally grasping something but that new knowledge leading to so many more questions. I was also a bit confused about the mechanisms driving apical constriction and your stepwise description really helped to clear this up for me so, thank you! Do you think that the formation of the hinge points is one of the most important parts of neurulation? Why or why not?

**Student A reply to student B:**

I like this question because it really forced me to think about the process of neurulation as a whole and what the goal of it is.. originally I would have said no. However, after thinking about how the point of all this is to get closure of the folds, I would say that hinge point formation is very important. I just might even say it is the most important step of neurulation because without it we wouldn't be able to close the neural folds, which would be detrimental.

**______________________________________________**

**Sample blog prompt from *Introductory Biology*:**

Some cancers run in families – that is, certain families have higher susceptibility to particular forms of the disease than others. Using your knowledge of gene inheritance, and how information in genes turns into cell function, explain why this is the case.

**Student A response:**

Because cancer is the result of mutations that disrupt the cell cycle, causing cells to grow unregulated, someone that already has a preexisting mutation in a proto-oncogene or tumor suppressor gene is more susceptible to acquiring it during their lifetime. Therefore, cancer tends to run in families. If someone inherits a mutation in a proto-oncogene or a tumor suppressor gene, then their cells will undergo promoted cell growth, allowing for them to acquire further mutations during their lifetime (at a faster rate than most other people), which could lead to cancer. Because the mutation to a proto-oncogene or tumor suppressor gene can be pre-existing ( or a mutation that occurs in a gamete being passed down), this mutation has a 50% chance of being inherited by the offspring; thus we tend to say that cancer runs in families.

**Student B comment to Student A’s response:**

Your response made me think of how some people will get tested to see if they have the cancer gene that their elder relatives did and as a result, they will have the possible site of cancer growth removed prior to any growths occurring. For women, some will have a mastectomy prior to having cancer because they have the BRCA1 or 2 mutation and do not want the risk of having cancer.
